# Significance and Roles of *Proteus* spp. Bacteria in Natural Environments

**DOI:** 10.1007/s00248-015-0720-6

**Published:** 2016-01-09

**Authors:** Dominika Drzewiecka

**Affiliations:** Department of General Microbiology, Institute of Microbiology, Biotechnology and Immunology, University of Łódź, 90-237 Łódź, Poland

**Keywords:** Natural microflora, Symbionts, Pathogens, Fecal pollution, Bioremediation, PGPR

## Abstract

*Proteus* spp. bacteria were first described in 1885 by Gustav Hauser, who had revealed their feature of intensive swarming growth. Currently, the genus is divided into *Proteus mirabilis*, *Proteus vulgaris*, *Proteus penneri*, *Proteus hauseri*, and three unnamed genomospecies 4, 5, and 6 and consists of 80 O-antigenic serogroups. The bacteria are known to be human opportunistic pathogens, isolated from urine, wounds, and other clinical sources. It is postulated that intestines are a reservoir of these proteolytic organisms. Many wild and domestic animals may be hosts of *Proteus* spp. bacteria, which are commonly known to play a role of parasites or commensals. However, interesting examples of their symbiotic relationships with higher organisms have also been described. *Proteus* spp. bacteria present in soil or water habitats are often regarded as indicators of fecal pollution, posing a threat of poisoning when the contaminated water or seafood is consumed. The health risk may also be connected with drug-resistant strains sourcing from intestines. Positive aspects of the bacteria presence in water and soil are connected with exceptional features displayed by autochthonic *Proteus* spp. strains detected in these environments. These rods acquire various metabolic abilities allowing their adaptation to different environmental conditions, such as high concentrations of heavy metals or toxic substances, which may be exploited as sources of energy and nutrition by the bacteria. The *Proteus* spp. abilities to tolerate or utilize polluting compounds as well as promote plant growth provide a possibility of employing these microorganisms in bioremediation and environmental protection.

## Introduction

### *Proteus* Like in Homer’s Poem…

**Fig. 1:**
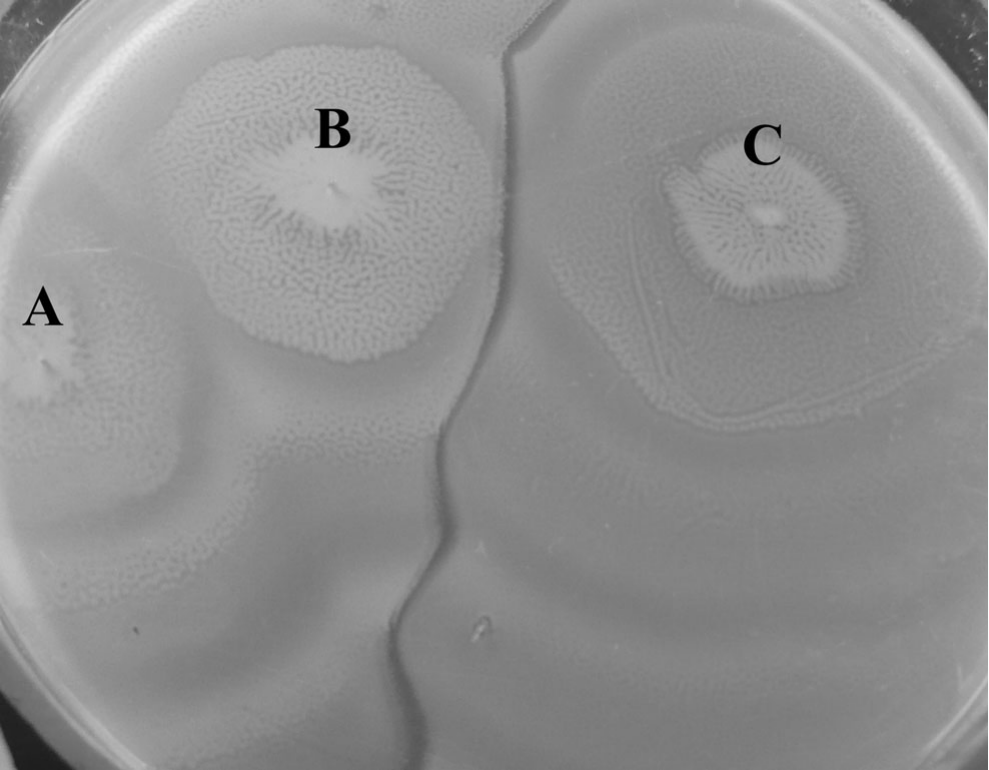
*P. mirabilis* strains swarming on the surface of an agar plate: isogenic A and B (no line of demarcation is visible) versus unrelated C (clear Dienes line of demarcation)

Microorganisms belonging to the genus *Proteus* were first described in 1885 by a German microbiologist Gustav Hauser, who had revealed their ability to swarm on solid surfaces. The name *Proteus* came from Homer’s “Odyssey” and its character Proteus, who could change his shape and had an ability of endless transformation. Hauser described two species of the genus: *Proteus vulgaris* and *Proteus mirabilis* [81]. The swarming ability, connected with a change of short swimmer cells into long, poli-nucleous and hyper-flagellated swarmer cells, is especially visible in the second species. This is a possible source of the name of *P. mirabilis*, which in Latin means amazing, marvelous, splendid. Hauser might have considered *P. vulgaris* to be more common and ordinary; therefore, he gave it the Latin name meaning widespread, usual. The ability of swarming growth is used in a simple and effective Dienes test to differentiate *Proteus* spp. strains with discriminatory power comparable to ribotyping [114]. The phenomenon described by Dienes in 1946 consists in forming boundaries between the swarming growth of different strains, while isogenic strains merge with each other (Fig. 1). However, the background of the expression of the territorial competition between two swarming non-isogenic strains still remains unclear [8, 48]. The formation of the boundaries (Dienes lines) may depend on different profiles of produced proticines (*Proteus* bacteriocines) and different profiles of strain sensitivity [129]. Budding et al. [19] suggested that one of the meeting strains dominated over the other killing it in the cell–cell contact because round dead cells were observed near the border in the swarm of the dominated strain. The Dienes reaction is connected with at least three recognized gene clusters enabling the self-recognition of *P. mirabilis* bacteria. Two of them (*idr* and *tss*) encode cytotoxins and a type VI secretion system, respectively [151], while the third one (*ids*) encodes the Ids proteins responsible for self-identification [21, 49]. However, the mutation in the *ids* genes does not provoke the killing of parent or mutant strain cells, although the Dienes line is visible between their swarms [48].

The genus *Proteus* includes Gram-negative, facultative anaerobic, heterotrophic, and proteolytic rods being human opportunistic pathogens. The taxonomic classification of these bacteria has changed several times. Lately, the only *Proteus* species with no clinical significance, *Proteus myxofaciens*, has been postulated to be moved from the genus *Proteus* to a new genus *Cosenzaea* [47]. Among other changes, the exclusion from the genus *Proteus* of several species that created new genera *Providencia* and *Morganella* is worth mentioning. These three closely related genera have formed the tribe *Proteeae* in the family *Enterobacteriaceae* [81]. Currently, the genus *Proteus* consists of *P. mirabilis*, *P. vulgaris*, *Proteus penneri*, *Proteus hauseri*, and three genomospecies 4, 5, and 6. *P. hauseri* as well as the genomospecies were separated from *P. vulgaris* on the grounds of molecular studies and form *P. vulgaris* group. The genomospecies 4, 5, and 6 are marked only with numbers as there have been no metabolic properties indicated to allow their full differentiation [104]. The main biochemical features typical of the genus and distinguishing *Proteus* species are summarized in Table 1.

**Table 1:** The main metabolic characteristics of *Proteus* spp. [103, 104]: “+”positive in 100 % strains; “−”negative in 100 % strains; in the other cases, the percentage of strains exhibiting positive reaction is given in parentheses

Feature	*P. mirabilis*	*P. penneri*	*P. vulgaris*	*P. hauseri*	*P.* genomospecies 4, 5, and 6
Typical of the genus and distinguishing it from the other *Enterobacteriaceae*					
Phenylalanine deaminase Lysine decarboxylation Arginine hydrolysis Urease production Glucose fermentation Mannose fermentation Lactose fermentation Methyl-red Growth in KCN Tyrosine clearing	+ (98 %) − − + (98 %) + − − (2 %) + (97 %) + (98 %) + (91 %)	+ (99 %) − − + + − − (1 %) + + (99 %) +	+ − − + (86 %) + − − + (86 %) + (99 %) +	+ − − + + − − + + +	+ − − + + − − + + (97 %) +
Typical of the species and allowing their differentiation					
Ornithine decarboxylation Indole production Lipase production DNase production Maltose fermentation Sucrose fermentation Salicin fermentation Esculin hydrolysis Citrate utilization	+ (99 %) − (2 %) + (92 %) +/− (50 %) − − (15 %) − − + (65 %)	− − − (35 %) − (12 %) + (96 %) + − − −	− + − (14 %) + + + + + − (29 %)	− + − − + + − − −	− + + (97 %) + (85 %) + + − (3 %) − (3 %) −

Bacteria from the genus *Proteus* can also be differentiated on the basis of their O-antigen variability, although serotyping is not included in the routine diagnostics of these rods. So far, there have been established 80 O-antigenic serogroups in the genus, some of them divided into subgroups [7, 67, 135], and many new O serotypes are still being discovered (D. Drzewiecka, unpublished data). The chemical structure of the sugar part of the lipopolysaccharide may play an important role in the adaptation of *Proteus* spp. bacteria to environmental conditions and enhancing their pathogenicity, as some O serotypes are more prevalent and more frequently isolated from clinical sources than the others (Table 2) [7, 35, 37, 72, 111, 133].

**Table 2:** Pathogenicity of *Proteus* spp. rods to humans [7, 8, 35, 37, 72, 81, 111, 125, 133, 148]

Infections	Virulence factors	Prevalent O-serotypes
• Urinary tract infections (cystitis, prostatitis, pyelonephritis, kidney stone formation) • Wounds and burns infections, abscesses • Respiratory tract infections • Bacteremia • Meningitis • Intestine colonization (diarrhea) • Nosocomial infections • Rheumatoid arthritis (?) – autoaggressive antibodies may arise due to the molecular mimicry between *Proteus* hemolysin and urease, and human white blood cells	• Fimbriae – adhesion • Flagella – swimming, swarming growth • Urease – urine pH elevation, kidney stones formation • LPS – endotoxin • O and capsular polysaccharides – swarming facilitation, kidney stones and biofilm formation, serospecificity • Biofilm – protection • Invasiveness – internalization into host cells • Haemolysins – cytotoxicity • *Proteus* toxic agglutinin – cell–cell aggregation, cytotoxicity • Proteases – antibodies degradation • Deaminases – α-keto-acid siderophores production and iron acquisition • Zinc and phosphate transport systems – utilization	• O3, O6, O10, O11, O13, O23, O24, O27, O28, O29, O30 – including *P. mirabilis* and *P. vulgaris* strains • O17, O61, O64, O65 – including *P. penneri* strains mainly • O78 – including *P. mirabilis* strains (data only from Poland) • O79 including *P.* genomospecies strains (data only from Poland)

*Proteus* spp. bacteria have been isolated from different human and non-human environments and their presence in higher organisms, soil, and water is well documented. However, their specific features and roles played in their natural habitats have not been summarized so far.

## Human Beings and *Proteus* Bacteria—Commensals and Parasites

*Proteus* spp. bacteria are mostly known as opportunistic human pathogens. Their roles in the pathogenesis of human beings as well as their virulence factors enabling the bacteria to reach different niches of the host organism and survive have been extensively studied and reviewed [8, 36, 81, 89, 103, 124, 125] and are summarized in Table 2. The bacteria cause infections mainly in people with an impaired immunity system, and most all of them may be a source of complicated urinary tract and wound infections as well as nosocomial infections. Urinary tract infections are most frequently ascending, often connected with the presence of urinary catheters. It should be noticed that *Proteus* spp. are the most prevalent bacteria, isolated from bladder and kidney stones (70 % of cases). *P. mirabilis* is the main cause of all *Proteus* spp. infections accounting for 80–90 % of them.

It is postulated that human intestines are a reservoir of *Proteus* bacteria, especially those belonging to prevailing *P. mirabilis* species, and they are members of natural fecal microflora of several percent of human population [124]. Lately, Porres-Osante et al. [118] reported the presence of *Proteus* spp. bacteria (one *P. mirabilis* and one *P. vulgaris* strain) in fecal samples from 4 % of healthy Spanish volunteers. Zilberstein et al. [160], using cultivation methods to study the microbiota in particular parts of the digestive tract, stated that *Proteus* spp. bacteria were absent from the mouth and the esophagus of healthy volunteers. However, the bacteria were present in comparatively high mean concentrations in the stomach of 8.1 % of volunteers (10^5^ colony forming units (CFU)/mL), the duodenum (45.5 %, 10^2^), the proximal jejunum (45.5 %, 10^4^), the proximal ileum (20 %, 10^6^), the distal ileum (19 %, 10^3.5^), the cecum (12.5 %, 10^4^), the colon (ascending, 33.3 %, 10^5^; transverse, 37.5 %, 10^5^; descending, 25 %, 10^5^; and sigmoid, 34.8 %, 10^7^), and in the rectum (30 %, 10^7^). The other members of the family *Enterobacteriaceae*—*Escherichia coli*, *Enterobacter* spp., and *Klebsiella* spp.—were detected in the same places in the lower digestive tract, but they were more prevalent.

*Proteus* spp. are regarded to be an undesired element of intestinal microflora, as the bacteria may also become a causative agent of diarrhea. Although Ikeobi et al. [59] did not notice significant differences in the presence of *Proteeae* members in the intestines of healthy individuals and diarrheal patients, Müller [91, 92] isolated *P. mirabilis* strains statistically more frequently from feces of patients suffering from diarrhea than from healthy individuals. The author suggested that the bacteria may become opportunists when the illness is caused by other intestinal pathogens or they may lead to intestinal disorders independently. Also, *P. penneri* strains were more frequently isolated from sick than from healthy people, while *P. vulgaris* were present in almost the same number of individuals in each group.

Thus, the presence of *Proteus* spp. bacteria in the gastrointestinal tract may also be treated as a carrier state, because in some conditions, it may lead to cross-infections and autoinfections especially in the urinary tract, which was observed many years ago by de Louvois [73] using serotyping as well as the Dienes test and confirmed in other studies [8, 48]. Chow et al. [26] emphasized the role of the intestinal tract as a reservoir of *P. mirabilis* bacteria leading to person-to-person transmitted nosocomial infection. Drzewiecka et al. [34, 35], by the use of serological and molecular methods as well as the Dienes test, proved that *P. mirabilis* strains isolated from feces and urine of patients were in fact the same spreading clone causing autoinfection and nosocomial infection. Wang et al. [148] reported the case of food poisoning in a restaurant in Beijing, China, caused by *P. mirabilis*. The same clone, identified by genotyping and the Dienes method, was detected in the consumed food and in stools of consumers as well as the cook handler and the waiter who, due to the lack of hygiene, most probably had transported the bacteria to the meal. The fact that dirty hands may be an important step in feces-to-hand-to-mouth spread of *Proteus* spp. bacteria was also confirmed by other researchers. Smith et al. [136] found *P. vulgaris* among the bacteria and yeasts isolated from roadside telephone receivers in Lagos, Nigeria. Padaruth and Biranjia-Hurdoyal [110] detected *Proteus* spp. on hands of primary school pupils in Mauritius while Shojaei et al. [132] on hands of food handlers in Iran (but simple washing resolved the problem). Also, Qadripur et al. [119] found *P. mirabilis* to colonize hand skin between nail plate and nail fold in motor mechanics.

Peerbooms et al. [113] compared *P. mirabilis* strains isolated from urine and feces, and all the isolates seemed to display similar virulence potential, which confirmed that the strains attacking the urinary tract may come from the intestinal reservoir. Also, Senior and Leslie [130] concluded that frequent isolation of *P. mirabilis* from feces and rare occurrence of *P. vulgaris* in the intestines of healthy individuals and those suffering from gastroenteritis may explain the fact that it is the first species and not the second one that is strongly connected with urinary tract infections.

The presence of *P. mirabilis* in the intestines may also be connected with obesity. Lecomte et al. [74], conducting research on an animal model, have recently reported on the changes in gut microflora depending on diet. Studying the intestinal microbiota of rats fed a high-fat diet (43 or 51 % of fat), the authors noticed higher numbers of *P. mirabilis* as compared to the control group (12 % fat diet). Moreover, a significant positive correlation was found between the abundance of *P. mirabilis* (as well as *Phascolarctobacterium* and *Veillonellaceae*) and all ten analyzed metabolic parameters associated with obesity.

## *Proteus* spp. in Animals—Adverse and Friendly

Not only rats but also many wild and domestic animals (mammals, birds, reptiles, amphibians, insects, and “seafood”) are the hosts of *Proteus* spp. bacteria. The relation of the bacteria to their host organism is sometimes not determined; in some cases, it may be symbiotic or change from neutral/commensal to parasitic (Table 3). The microorganisms are an element of animal pathogenic or physiological microflora, especially in the intestines—a reservoir of the bacteria.

**Table 3:** Different kinds of relationship between *Proteus* spp. and other organisms (details in the text): “+” positive (beneficial), “?” neutral/commensal/not determined, “−” negative (antagonistic, pathogenic)

organisms	*Proteus* sp.	*P. mirabilis*	*P. vulgaris* group	*P. penneri*	selected references
Humans		? / −	? / −	? / −	8, 36, 81, 103, 124, 125, 148
Gorillas		?	?		15
Dogs	−	? / −	?	?	38, 44, 69, 99, 144
Cats, feral cats	−	−			54, 69, 99
Pigs		?	?		68, 75, 147
Horses	? / −				84, 152
Donkeys	−				83
Cow, cattle, calf		?	? / −	?	2, 56, 76, 123, 138
Raccoon dog			?		66
Flying fox		+	+		6
Rats		?			73
Birds, poultry	− (eggs)	?/−	? / −	? / −	9, 43, 46, 61, 62, 68, 71, 123, 154, 155, 161
Snakes		?	?	?	16, 53, 131
Alligator	−				101
Turtles	−	?	? / −	? / −	5, 10, 42, 53, 102, 108, 127
Amphibians			?		53
Fishes	+ / ?	+	+ / ? /−	+	17, 66, 70, 97, 107, 141
Oysters		?			39
Shrimps	+ / ?	+ / ?	+ / ?	+ / ? / −	20, 82, 97, 98
Lobsters	+	+	+	+	97
Blue crab				?	109
Sponges		+/?	?		50, 64
Millipede		+			4
Lepidopteran			−		85
Cockroaches		?	?		142, 145, 149
Honey bees	?				137
Flies	?	? / +	?		18, 51, 79, 80, 85, 87, 94, 134, 144, 150
Mites			+		2
Nematodes				−	76
Leguminous plants			+		12, 14, 120
Wild grass		+			121
Tea			+		12
Cabbage			+		156
Maize		+			60
Mould fungi	−		−		11, 12
*C. albicans*		−			50
*B. bacteriovorus*				−	20

*P. mirabilis* and *P. vulgaris* were found in fecal samples of western lowland gorillas (*Gorilla gorilla gorilla*), collected at two locations in south-central Cameroon, proving to inhabit the intestines of these great wild apes [15]. Like in humans, the presence of *Proteus* spp. in animal intestines may pose a threat of autoinfection and cross-infection. The example of such autoinfection was described by Gaastra et al. [44], who in the Netherlands isolated *P. mirabilis* strains from feces and urine of dogs suffering from recurrent urinary tract infections. It is probable that the intestine was a reservoir of the bacteria infecting the urinary tract of the dogs, because *P. mirabilis* strains were not isolated from feces of healthy controls. Also, Kroemer et al. [69] reported *P. mirabilis* strains as a reason for urinary tract infections in dogs and cats in European countries. Normand et al. [99] indicated *Proteus* spp. isolated from different materials (mostly feces, urine, skin swabs, upper respiratory tracks) as an important cause of illness in dogs and cats examined in Glasgow, UK. Moreover, 26 % of the strains were identified as multiple drug resistant. *P. mirabilis* was isolated from the rectum, the vagina, the mouth, the nose, and wound/abscess of feral cats analyzed in Grenada, West Indies [54]. However, all the isolates were sensitive to most of the used antibiotics, so it was concluded that feral cats did not pose a risk to humans or other cats. Kitamikado and Lee [66] isolated from feces of raccoon dog *Nyctereutes procyonoides*, a *P. vulgaris* strain producing chondroitinase, which may be regarded as one of the virulence factors produced by the microorganism because chondroitin sulfate is distributed in animal connective tissues.

On the other hand, there was no connection found between *Proteus* spp. and ulcerative keratitis [146] or transmissible venereal tumor [38] in dogs, as *P. penneri* and *P. vulgaris* strains were isolated from the eyes of healthy dogs in Beijing, China, and, respectively, *P. mirabilis* from the vagina of a healthy dog in Nigeria, but not from sick animals.

*Proteus* spp. bacteria may also be the members of natural microflora of the esophagus [84] and the skin of horses, although they were also isolated from wounds [152]. However, the wound isolates displayed strong adherence and significantly stronger attachment than skin isolates proving enhanced virulence of pathogenic strains, as compared to normal skin isolates. The bacteria are virulent also to donkeys causing urinary tract infections. They were found to account for 6.7 % of donkey uropathogens isolated in Ethiopia, among which *Streptococcus* spp. (43.3 % isolates) and *E. coli* (20 %) were dominating [83].

*P. mirabilis* and *P. vulgaris* were also detected as the elements of the microbial community in pigs. Lowe et al. [75] revealed that the family *Enterobacteriaceae* was a minor but significant component of pig tonsil microflora in which *P. mirabilis* or *P. vulgaris* clones dominated. Wang et al*.* [147] reported a *P. vulgaris* strain isolated from nasal swab of a pig from a food producing animal farm (sic!) that was carrying a chromosomally located staphylococcal multiresistance *cfr* gene encoding the resistance to linezolid, but also to other chemically unrelated classes of antimicrobial agents. The gene was for the first time reported in a naturally occurring Gram-negative bacterium. Still Kobashi et al. [68] isolated *P. mirabilis* strains from pig feces detecting the efflux genes responsible for their resistance to tetracycline (*tetH* and *tetJ*).

All *Proteus* species were detected in the cow. Hawkey et al. [56] revealed that *P. vulgaris* (including the strains currently numbered among genomospecies) as well as *P. mirabilis* species were, respectively, the first and the third species among the tribe *Proteeae* most commonly isolated from beddings contaminated with feces and urine in two calf farms in South West England. The authors concluded that the high similarity of the O-serotype profile of isolated strains (e.g., serotypes O23 and O30) to those reported for human infections (Table 2) suggests that food animals may be a source of *Proteeae* strains carried in human gut. Lu et al. [76] reported on the isolation from a cow dung in China of *P. penneri* strains displaying strong nematicidal activity. *P. vulgaris* seems to belong to normal skin microflora of cattle, but the involvement of these bacteria in the damage to tissues around the skin lesions caused by *Demodex bovis* mite and their synergistic influence was also revealed [2]. The symbiosis between the parasites relies on the fact that the mite introducing the bacteria into the skin on the exoskeleton or in the gut receives suitable microclimate for the establishment and replication due to many virulence factors and enzymes produced by the cooperating microorganisms.

Rogers [123] suggested a possibility of the transmission of potentially pathogenic bacteria, including *Proteus* spp., between wild birds and cattle. The author stated the presence of *P. vulgaris* in feces of 13 % dairy cattle in five studied farms, and in fecal and cloacal samples from 7.8 % analyzed birds (sparrows, blackbirds, cowbirds, but not starlings).

Other authors also emphasized the role of wild birds in the transmission and spread of pathogenic bacteria to domestic poultry, cattle, or humans, resulting in the change of their status from bird fecal microflora members to the etiological agent of pathogenesis. Yong et al. [155] found *P. mirabilis* to be prevalent in feces of large-billed crows (*Corvus* spp.) inhabiting the surroundings of a minimarket in Bangsar, Malaysia, and becoming a health hazard due to their large numbers. However, no bacteria (with the exception of *Klebsiella* spp.) were present in the air samples from the market place. Winsor et al. [154] conducted studies on fecal microflora of apparently healthy wild turkey vultures (*Cathartes aura*) in Texas, USA, because the diet of these birds, which are carrion-feeding animals, must include animals that have died of infectious diseases. The content of the studied bird intestines was dominated by *E. coli* but, in fact, *P. mirabilis* was the second predominant species detected in 50 % birds and *P. vulgaris* was isolated from one bird. Jahantigh [61, 62] stated that *Proteus* spp. strains in 2010 accounted for 5 % and in 2012 for 12.5 % isolates from eggs of the ostrich (*Struthio camelus*). The authors indicated fecal contamination that may lead to the penetration of the bacteria into the egg interior and the infection which may be a reason for a relatively high ratio of embryonic mortality in ostrich eggs in Iran. Awad-Alla et al. [9] suggested a possible role of white ibises (*Nipponia nippon*) in Egypt in the transmission of some pathogens to poultry as in the internal organs of apparently healthy birds they found several strains classified as *P. mirabilis* (although the indicated ability of indole production suggests that the strains should be identified as *P. vulgaris*; see Table 1). Next, Foti et al. [43] conducted studies on the health status of birds belonging to several orders migrating to Africa through the Ustica Island (Italy). From fecal swabs and internal organs of birds found dead, they isolated only one *P. mirabilis* strain, so the probability of dissemination of these bacteria was very low. Kwiecińska-Piróg et al. [71] revealed that *P. mirabilis* naturally occurring in the crop and the cloaca of white stork (*Ciconia ciconia*) healthy nestlings in Poland were more susceptible to antibiotics than clinical strains isolated from human beings and no ESBL production was detected.

The above data indicate that the presence of *Proteus* spp. in birds does not pose a real risk of dissemination; moreover, *Proteus* spp. bacteria seem to be a member of normal poultry microflora. Among the bacteria inhabiting the beak cavity and the cloaca of reproductive goose flocks (190–800 birds) from 17 farms in Poland, *Proteus* spp. colonized approximately 10–25 % of birds [161], while *E. coli*, *Enterococcus* spp., and *Streptococcus* spp. were found in approximately 60–70 % of birds and coagulase-negative *Staphylococcus* was isolated from 80–90 % birds, independently of the sampling site. Other bacteria were isolated sporadically. Also, Kobashi et al. [68] isolated *P. mirabilis* and *Proteus* sp. strains from poultry feces, detecting the *tetM* gene, encoding their resistance to tetracycline. However, Giacopello et al. [46] recognized antibiotic-resistant *P. mirabilis*, *P. vulgaris*, and *P. penneri* present in feces of sick domestic canaries as pathogenic microflora.

*Proteus* spp. colonize also amphibians and reptiles, e.g., *P. vulgaris* is reported as a common inhabitant both in the oral cavity and in the cloaca of water amphibians (*Lissotriton vulgaris* newts and *Pelophylax ridibundus* frogs) and reptiles (*Mauremys rivulata* turtles and *Natrix natrix* grass snakes) inhabiting the Kavak Delta, Turkey [53]. In the oral bacterial flora of Chinese cobra, *Naja atra*, and bamboo pit vipers, *Thimeresurus albolabris*, two snake species common in Hong Kong [131], *P. penneri*, *P. vulgaris*, and *P. mirabilis* strains were isolated among many other potentially pathogenic bacteria. Blaylock [16] reported on the common isolation of *Proteus* sp., *P. mirabilis*, and *P. vulgaris* from oral swabs of four different house snake species from southern Africa. There is also a report on pathogenic *Proteus* sp. as a cause (together with *Morganella morganii*) of the septicemia and death of a captive alligator held in the Savannah River Ecology Laboratory in Aiken, SC, USA [101].

There are several reports available concerning the presence of *Proteus* spp. bacteria in water turtles, sometimes connected with illness. They were the fourth genus most frequently isolated from lesions of sea turtles from the Canary Islands, Spain, and were considered as one of the causes of their diseases and mortality [108]. Al-Bahry et al. [5] interpreted the presence of *Proteus* spp. and *P. vulgaris* (resistant to ampicillin, streptomycin, and tetracycline) in oviductal fluids of nesting sea green turtles (*Chelonia mydas*) as an indicator of pollution in the surrounding areas between the Gulf of Oman and the Arabian Sea (see the next paragraph). In contrast, in nesting green turtles from Costa Rica, *P. mirabilis* and *P. vulgaris* bacteria were recognized as a non-pathogenic constituent of microflora. They were isolated from nasal and cloacal swabs from apparently healthy turtle females with no signs of disease. There was no correlation observed between the turtles and seawater bacteria composition, so it was suggested that *Proteus* bacteria should be treated as the physiological microflora of green turtles [127]. *P. mirabilis* was also commonly found as inhabiting the cloaca of European pond turtles (*Emys orbicularis*), especially captive ones, from Poland [102]. *P. vulgaris* was the predominant species, and some *P. mirabilis* strains were also identified in cloacal and oral swabs of loggerhead sea turtles (*Caretta caretta*) inhabiting the Sicilian Channel, the South Tyrrhenian Sea, and the Ionian Sea [42]. The loggerhead turtle is included in the Red List of the world conservation union, and the microbial contamination of the turtle eggs is suspected to play a role in embryonic death and a low loggerhead hatch success rate in Georgia, USA. Indeed, among other Gram-negative isolates from unhatched eggs of the turtle on Jekyll Island, Georgia, *P. penneri* and *P. vulgaris* strains were identified [10].

Although *Proteus* spp. bacteria are commonly known as opportunistic pathogens, there can be found interesting examples of positive relations between the microorganisms and the host animal (Table 3). *P. mirabilis* and *P. vulgaris* strains isolated from the intestine of the Indian flying fox (*Pteropus giganteus*) were recognized as the members of symbiotic physiological microflora of this big fruit bat [6]. Although this is unusual for the two species (Table 4), the isolates were able to produce cellulolytic and xylanolytic enzymes just as three other isolated species, *Citrobacter freundii*, *Serratia liquefaciens*, and *Klebsiella oxytoca*, and contrary to the other six gut isolates unable to digest cellulose. Fruits and leaves, which are the animal’s main food, are built of up to 50 % of cellulose, hemicellulose (xylan), lignocellulose, and pectin cellulose, and because mammals do not possess proper enzymes to degrade the polymers, the symbiotic enterobacteria play an essential role in their nourishment and digestion. Another example of a cellulolytic and xylanolytic *P. mirabilis* strain isolated as a gut symbiont of millipede (*Xenobolus carnifex*) was reported by Alagesan et al. [4].

**Table 4:** Unusual physiological features displayed by *Proteus* spp. strains isolated from different habitats (details in the text)

Feature	*Proteus* sp.	*P. mirabilis*	*P. vulgaris*	*P. hauseri*
Heterotrophic nitrification		Coastal seawater		
Cellulose digestion		Flying fox, millipede	Flying fox	
Lipase production/hydrocarbons utilization (including aromatic ones)	Contaminated soil; waste sludge	Contaminated soil	Contaminated soil; contaminated fish	
Phenol utilization		Contaminated soil		
Methyl tert-butyl ether (MTBE) degradation		Contaminated soil		
ɛ-Caprolactam utilization		Contaminated soil		
Hexachlorocyclohexane (HCH) pesticide utilization	Contaminated soil			
Phorate pesticide utilization	Contaminated soil			
Chlorpyrifos, methyl parathion, and p-nitrophenol pesticides degradation	Contaminated soil (in consortium)	Contaminated soil (in consortium)		
DDT reduction			Mouse	
Azo dyes decolorizing	Waste site	Contaminated soil; wastewater sludge		Hot spring
Phosphate solubilization	Phorate contaminated soil	Wild grass rhizosphere		
Copper tolerance		Wild grass rhizosphere; wastewater	Soil	Hot spring
Chromium,cobalt, cadmium, zinc,mercury, nickel, lead, arsenic tolerance		Wild grass rhizosphere; wastewater; contaminated soil		
Silver tolerance		Wastewater		
Chromate tolerance	Contaminated seawater			
Thermotolerance				Hot spring
Halotolerance	Salt lake	Oysters	Halophyte glasswort rhizosphere	
Acidotolerance	Soil contaminated by hydrocarbons		Acidic soil	Hot spring

Also, a symbiotic role has been suggested for *P. mirabilis* and *P. vulgaris* isolated from hematophagous sand fly *Phlebotomus papatasi* being a vector of *Leishmania* parasite, as the bacteria were the most prevalent in larvae, pupae, and mature male and female insects gut [80]. Another very interesting hypothesis indicating a close relationship between *P. mirabilis* and its blowfly host *Lucilia sericata* was formed by Ma et al. [79]. These proteolytic microorganisms are able to produce volatile components, for example, putrescine (from ornithine) and ammonia (see Table 1), important for their swarming ability and, at the same time, attracting flies to the carcass. The authors stated that putrescine, which is an extracellular signal required for the swarming phenomenon and used by *P. mirabilis* in *quorum sensing* [89], may be an interkingdom signal sensed by both the insects and the bacteria. The same role was suggested for ammonia. Both compounds as well as four other attractants (NaOH, KOH, phenol, and lactic acid) restored the swarming motility in different swarming-deficient mutants of the *P. mirabilis* strain isolated from maggot salivary glands where they dominated, so the researchers linked fly attraction with *Proteus* swarming. The bacteria were detected also in adult *L. sericata* flies and in *Lucilia cuprina* sister species [134]. The symbiosis between blowflies and *Proteus* bacteria is also indicated by the fact that *P. mirabilis* biofilm, contrary to biofilms constructed by *Staphylococcus aureus* and *Enterobacter cloacae*, is not disrupted or is even stimulated by *L. sericata* maggots used in the debridement therapy. Simultaneously, *P. mirabilis* is antagonistic to some bacteria eliminated by maggots from wounds, protecting the larvae from harmful microorganisms [18]. Complete sterility of *L. cuprina* maggots for wound therapy was achieved in all cases except for *P. mirabilis* [87]. Additionally, Wei et al. [150] revealed that both sensitive and antibiotic-resistant *P. mirabilis* strains could persist for several days among the gut microflora of the green bottle fly (*L. sericata*) or the housefly (*Musca domestica* L.) when introduced by feeding. It has been speculated that the mechanisms stimulated by the fly host may contribute to the maintaining of antibiotic-resistant strains in particular, and in that way, their transmission is imminent. It was also suggested [144] that flies, as the vectors of bacteria to raw meat devoted to dog breeding, were responsible for a high percentage of morbidity and mortality due to intestinal infections among pups in greyhound dog kennels in Kansas, USA. A high percentage of bacterial contamination among blowflies (different species) and domestic flies (*M. domestica*), stable flies (*Stomoxys calcitrans* L.), flesh flies, and others was observed, while *Proteus* spp. were proved to be the most common bacteria among Gram-negative and lactose-negative ones isolated from flies, followed by *Providencia* spp., *Pseudomonas* spp., and *Salmonella* spp. Nazni et al. [94] identified *Proteus* as the second (after *Enterobacter*) dominating genus on the external body surface, while in the fly gut, *Proteus* spp. dominated among the bacteria isolated from the housefly (*M. domestica*) on a poultry farm in Malaysia. *P. mirabilis* with *Providencia* spp. was also prevalent in gut microflora of flies captured in different public places in India [51].

Not only flies but also cockroaches are regarded as common vectors of different microorganisms, including *P. mirabilis* and *P. vulgaris* strains, carrying them on their bodies and posing a threat of their dissemination, food contamination, and spoiling as well as infection of humans. The bacteria were found in Nigerian cockroaches in Ekpoma, a village characterized by poor sanitary conditions [142], and in Iranian brown-banded cockroaches collected in kitchens of Ahvaz houses [145]. Wannigama et al. [149] reported on the isolation of *P. mirabilis* from 8.9 % cockroaches found in households and food-handling establishments in Varanasi, India.

*P. vulgaris* was isolated from tissues of an American fly *Drosophila paulistorum* [85], although it was not determined if the microorganisms were parasitic, mutualistic, or symbiotic to their host. However, they displayed strong pathogenicity toward lepidopterans like *Heliothis virescens*. Other insects which may carry *Proteus* spp. bacteria in the intestines are bees [137]. It is suggested that the source of the bacteria is pollen consumption and bees in the colony are infected one by one during food exchange. Honey pollution by *Proteus* spp. may pose a threat to consumers.

A similar situation may be observed in water animals, so called “seafood,” where the presence of *Proteus* spp. may result in food spoiling and poisoning, e.g., scombroid poisoning of fish meat as a result of histidine decarboxylation leading to a rise in the level of toxic histamine [100]. A source of bacteria, including antibiotic-resistant ones, may be fecally contaminated water (see the next paragraph), because *P. vulgaris* and *P. mirabilis* rods were found as absorbed on body structures of the commercially important sponge *Spongia officinalis*, an animal inhabiting the Aegean Sea and feeding by the seawater filtration [64]. Also, Graça et al. [50] reported on the isolation of several *Proteus* sp. strains (closely related to *P. mirabilis*) from the marine sponge (*Erylus deficiens*) collected 150 km off the southwest coast of Portugal. The authors revealed that *Proteus* sp. strains (together with *Pseudoalteromonas* and *Microbacterium* spp. isolates) presented the strongest bioactivity against pathogenic bacteria and *Candida albicans*, thus protecting the host animal and assisting its survival, also due to the fact that the absorbed bacteria constitute 50–60 % of the sponge biomass.

However, the occurrence of these opportunistic human pathogens in oysters poses a health risk if the shellfish are consumed raw. Fernandez-Delgado et al. [39] found halophilic (growth in saline concentrations from 3 to 8 %) *P. mirabilis* as prevailing in the bodies of two oyster species in Venezuela and resistant to several tested antibiotics (mainly tetracycline, ampicillin and penicillin, and cefoxitin and cefazolin). Also, Matyar et al. [82] isolated several antibiotic-resistant *P. vulgaris* and *P. penneri* strains from the intestines of shrimps inhabiting the Iskenderun Bay, Turkey, which was most probably due to the Iskedrun Bay contamination by industrial and domestic wastes, including hospital ones. It is worth noting that *Proteus* spp. strains were absent from seawater and sediments, although high amounts of fecal coliforms indicated strong fecal contamination of the Bay. Nimrat et al. [98] identified *Proteus* spp. and *P. mirabilis* among other bacteria detected in spermatophores from black tiger shrimps (*Penaeus monodon*) collected from the Andaman Sea, Thailand. Cao et al. [20] recognized *P. penneri* as an agent causing red body disease of commercial white shrimps *Penaeus vannamei*, cultivated in Xiaoshan, Zheijang, China. Interestingly, the mortality of shrimps was successfully inhibited by the predatory activity of *Bdellovibrio bacteriovorus* against the *P. penneri* pathogen; thus, the authors propose this bacterial predator as a potential biocontrol agent. *P. penneri* strain inter alia was also detected on the body surface of the wild blue crab (*Callinectes sapidus*) in the Akyatan Lagoon (the south of the Mediterranean Sea) [109].

*Proteus* spp. rods are found in marine fishes. In Atlantic mackerel (*Scomber scombrus*), *P. vulgaris* and *Proteus* sp. were detected in gills, skin, and gut as the only members of the family *Enterobacteriaceae* [141]. In *Scomber japonicus* mackerel (the intestine and the stomach) or in *Limanda herzensteini* flat fish (the gills), *P. vulgaris* was stated as producing a big yield of intracellular and extracellular chondroitinase, which may be directed against the host connective tissues, containing mucopolysaccharide chondroitin [66]. Also, freshwater Nile tilapias (*Oreochromis niloticus*) from experimental freshwater aquaculture in Brazil [17] and tilapias from Lake Victoria, Kenya [107], were sporadically colonized by *P. vulgaris* or *Proteus* sp. Kumar et al. [70] reported on *P. hauseri* as a causative agent of hemorrhage and mortality in a koi carp (*Cyprinus carpio*) farm in India. This is the first animal habitat reported for this poorly recognized species, while the sources of isolation of two previously described *P. hauseri* strains (one human) remain unknown [104] and two other strains were isolated from human urine [63].

The presence of *Proteus* spp. bacteria was also revealed to have a surprisingly positive influence on water animals. Many *P. mirabilis*, *P. vulgaris*, *P. penneri*, *P.* genomospecies 4, and unidentified *Proteus* sp. strains isolated from the intestines of black tiger shrimp, cobia marine fish, snubnose pompano marine fish, and ornate spiny lobster in Vietnam demonstrated probiotic properties linked to bacteriocin production and their antagonistic activity towards many pathogenic bacteria [97].

## *Proteus* as an Indicator of Fecal Pollution

The presence of *Proteus* spp. bacteria in water and soil may indicate the fecal pollution of the environments where these proteolytic bacteria are treated as allochthonic. Human and animal feces are probably an important source of these rods in natural environments. Water animals may absorb pollutant microorganisms from water. There is a potential risk of their spread in the marine food chain as well as dissemination during the food processing and transmission to humans after consumption. For instance, bacteria (in that number *Proteus* spp.) associated with sponges (*S. officinalis*), which are the main filter feeders in marine environment, were proposed to be treated as indicators of fecal contamination of marine ecosystems [64]. Other marine animals that are reported to accumulate *Proteus* spp. from water environment are oysters [39], loggerhead turtles (*C. caretta*) [42], and green turtles (*C. mydas*) [5], although Santoro et al. [127] did not find the correlation between green turtle microflora and seawater microorganisms.

Also, antibiotic-resistant *Proteus* spp. strains may be released from the intestines which are a source of drug-resistance genes [126]. Some authors [5, 39, 82] emphasize the fact that marine environment seems to be a reservoir of genes responsible for the antibiotic resistance of polluting bacteria, as many antibiotic-resistant strains, including *Proteus* spp., are isolated from water and sea animals (see the previous paragraph). A good example is also the Jiaozhou Bay on the western coast of the Yellow Sea, China, which is highly contaminated due to intensive industrial development and urbanization. Effluents from hospitals and wastewater may be the sources of drug-resistant bacteria, allowing the resistance genes transfer to environmental microflora. Many bacterial strains resistant to tetracycline or chloramphenicol, including *P. mirabilis*, were isolated from the seawater by Dang et al*.* [28, 29]. Also, in Cameroon studies of water samples collected for a period of 8 months from Douala Lagoon, contaminated by industrial and domestic wastes, resulted in the isolation of *P. vulgaris* strains among other fecal bacteria in each sample, which poses a serious health problem [3].

The fact of *Proteus* spp. survival in marine environments indicates the ability of these microorganisms to adapt to higher salinity conditions, similarly to the strains from oysters, mentioned earlier. Indeed, several halotolerant *Proteus* spp. strains were detected in water samples from El Golea Salt Lake, Algerian Sahara, proving that domestic wastes from the El-Goela oasis could be a source of the fecal microflora surviving in these conditions [52].

*Proteus* spp. can also be found in freshwater, as an indicator of its contamination by feces, even in unexpected habitats. Microbiological investigation of water and sediments from the Vrelo Cave, the Republic of Macedonia [33], revealed mostly the presence of *Bacillus* spp. strains (83 %), but, additionally, allochthonic strains were detected, including *P. penneri*, in a water sample taken from a place located 400 m from the cave entrance and 100 m deep. Moreover, although physical, chemical, and biochemical parameters indicated a high water quality, big numbers of total and fecal coliform bacteria were observed both in water and in sediment samples, suggesting their contamination from an animal source.

The detection of bacteria belonging to the genus *Proteus* in drinking water disqualifies its suitability for consumption due to its fecal pollution, which would threaten with waterborne infections. The problem occurs in India. *P. vulgaris* was claimed to be the main microbial pollutant of drinking water in Rajasthan [140]. Then, in bore well waters in Mysore City, *P. mirabilis* and *P. vulgaris* strains dominated over the other H_2_S-producing strains (considered as associated to fecal coliforms in drinking water) [93]. Poonia et al*.* [117] reported on the alarming presence of multidrug resistant *P. mirabilis* and *P. vulgaris* strains among other bacteria in drinking water from springs and streams in the rural areas of Sikkim. Also, in Nigeria, *Proteus* spp. bacteria were detected in two of five studied well waters, treated as a source of drinking water [1].

*P. mirabilis*, *P. vulgaris*, and *P. penneri* strains, in addition to other members of *Enterobacteriaceae*, were recognized as bacteria connected with an anthropic influence, indicating the fecal contamination of rice field water from irrigation channels and rice field plots in Rio Grande do Sul, Brazil [122].

Also, in soil, the presence of *Proteus* spp. bacteria is regarded as the evidence for its fecal contamination. Srinivasan et al. [138] reported on the isolation of *P. mirabilis* strains from the soil samples from a dairy farm area that was regularly treated with cow manure, but not from the control forest soil samples with no history of agriculture. Trawińska et al*.* [143] detected both *Proteus* spp. and *E. coli* strains in soil samples, collected near or 300 m from the reproductive layer farm in Poland, although the grounds were not treated with manure or exploited. Thus, it was suggested that the bacteria associated with fecal contamination of soil may have come from other animals, because the soil samples also contained *Toxocara* spp. eggs, which are not found in birds.

## *Proteus* in Bioremediation and Plant Growth Promotion

*Proteus* spp. bacteria, as an element of intestinal microflora of humans and animals, are often treated as zymogens in soil or water. In fact, these proteolytic microorganisms come to these environments with feces or waste, and after digestion of approachable organic matter, they decay due to the lack of nutrients.

However, these microorganisms are also found in such habitats as autochthones, well adapted to the environmental conditions, exhibiting unusual and exceptional metabolic features (Table 4), although this aspect of the genus *Proteus* lifestyle is less known. An interesting example is a *P. mirabilis* strain isolated from coastal seawater in China, which was characterized as a heterotrophic nitrifier [158]. The strain effectively removed ammonia (NH_4_^+^) ions by oxidation. Moreover, only trace amounts of NO_2_^−^ and NO_3_^−^ were detectable after cultivation as the ions were simultaneously reduced by the bacterium to gaseous nitrogen (N_2_) in the denitrification processes. The processes and the cell growth were inhibited in the absence of any organic source of carbon. The strain is suggested to be used for toxic NH_4_^+^-N removal, although its activity in oligotrophic water was limited probably due to the lack of carbon sources. Similar metabolic activity was displayed by the *Proteus* sp. strain related to *P. mirabilis*, isolated from effluents from a fish processing plant in India [86]. Simultaneous aerobic nitrification and denitrification leading to the efficient removal of nitrogen by this heterotrophic bacterium are suggested to be applied in fish waste treatment.

*Proteus* spp. bacteria, though well known as opportunistic pathogens, in natural environments show more positive aspects of their existence. They may play a role of effective and specialized plant-growth-promoting rhizobacteria (PGRP) or bioremediators of hydrocarbons, pesticides, herbicides, aromatic compounds, azo dyes, and heavy metals in contaminated environments.

Lipase production is not typical of all *Proteus* species (Table 1), but the rods in natural environments are able to effectively degrade hydrocarbons, including oils, and to remove these hazardous substances efficiently and inexpensively. Kim et al*.* [65] reported on a *P. vulgaris* strain producing extracellular alkaline lipase isolated from soil samples collected near a sewage disposal plant in South Korea. The lipase was stable from pH 5 to 11 and had a maximum activity at pH 10. Lu et al*.* [77] isolated from soil in China a *P. vulgaris* strain which produced alkaline non-position-specific lipase, whereas Whangsuk et al*.* [153] found in a waste-sludge from beer factory in Taiwan a *Proteus* sp. strain actively giving off organic-solvent-tolerant lipase named LipA, which effectively conversed palm oil into biodiesel.

The first report on a *Proteus* sp. strain which could be useful as a sole degrader of oil hydrocarbons in soil was presented by Hernandez-Rivera et al*.* [57]. The strain may be presumed to belong to *P. vulgaris* group due to indole production and maltose fermentation abilities observed by the authors (see Table 1). It was found in tropical soil containing total petroleum hydrocarbons (TPHs) in the Tabasco region, Mexico, highly contaminated by oil spills during 20 years of pollution and was able to remove the superficial hydrocarbon layer (the only carbon source) in the culture medium forming a stable emulsion, most probably due to the production of biosurfactants by the strain itself. The results were better than in the medium containing urea (actively utilized by the bacteria; see Table 1). Then, Ibrahim et al*.* [58] studying soil samples collected from the rhizosphere of legumes planted on crude-oil-contaminated soil in Kaduna, Nigeria, found *P. mirabilis* and *P. vulgaris* strains as belonging to the most active crude oil degraders among the several isolated species, although not displaying biosurfactant production. Earlier, another hydrocarbon degrading *P. vulgaris* strain had been isolated from newly killed fish samples that were collected near the point of spill in the Niger Delta, where most of the crude oil in Nigeria is found. The isolated strain was able to utilize Bonny light crude oil, diesel, and kerosene, generating organic acids. The finding is promising for this region, where the oil spills are a source of significant air, soil, and water pollution, destroying biodiversity in the ecosystem [105]. Lutz et al*.* [78] described a wild-type bacterial cocktail marketed as Superbugs^®^ and composed of bacteria belonging to genera *Proteus*, *Bacillus*, *Pseudomonas*, *Citrobacter*, and *Enterobacter*, as effective in the biodegradation of ethyl biodiesel made from palm triacylglycerols in Costa Rica.

There are several reports on *Proteus* spp. strains able to biodegrade polyaromatic hydrocarbons (PAHs). The genus *Proteus* dominated after *E. coli* in the group of 60 different bacterial strains able to degrade hydrocarbons, classified as hazardous waste in soil (benzene, toluene, octane, heptanes, biphenyl, naphthalene, camphor, and phenanthrene), isolated from soil samples near different petrol pumps of Karachi City, Pakistan [139]. The genes responsible for the hydrocarbon degradation ability were located either chromosomally or extra-chromosomally. The plasmid location is promising for bioremediation processes since the genes can be conjugated to other microorganisms in polluted environments. Ceyhan [22] reported on a *P. vulgaris* strain, isolated from biofilm in wastewater of the petrochemical industry in Turkey, which was effective in the degradation of pyrene (four-ring PAH) as a sole source of carbon and energy. Moreover, the degradation of this highly toxic and carcinogenic hydrocarbon resulted in non-toxic and non-accumulating metabolites, proving a big biodegradation potential of the strain.

Gasoline-contaminated soil from a gas station in Chihuahua, Mexico, was the habitat of a *P. mirabilis* strain degrading with medium efficiency methyl tert-butyl ether (MTBE), which is a toxic synthetic compound added to gasoline in a high concentration of 15 %, as a blending component [90]. Next, a *P. mirabilis* strain was found, which was able to efficiently degrade phenol as a sole carbon and energy source in an oil-contaminated soil sample collected in India [88]. Sanuth et al*.* [128] identified a *P. mirabilis* strain which was the most effective in ɛ-caprolactam degradation among the bacteria isolated from soils collected from the major solid waste dumpsites in Lagos State, Nigeria. ɛ-Caprolactam is a monomer for nylon-6 production, found in wastewater effluents from nylon-producing factories, toxic to plants, animals, and humans. The strain seems to be a potential candidate for its bioremediation.

Bacteria from the genus *Proteus* solely or in consortia also display an ability to neutralize different toxic herbicides and pesticides that may cause heavy pollution in terrestrial and aquatic ecosystems, especially when inappropriately used. *P. vulgaris* from a mouse intestine was one of the first reported bacteria solely able to reduce dichloro-diphenyl-trichloroethane (DDT) pesticide to dichloro-diphenyl-dichloroethane (DDD) [41]. Although DDD is also a toxic pesticide, its production is the first step during the degradation and mineralization of DDT, and it is utilized in further transformations.

Correa and Steen [27] found a *P. mirabilis* strain to be the fastest degrader of the propanil (a commonly used herbicide) among the natural microflora inhabiting a pristine lake in northeast Georgia, USA, followed by the bacteria belonging to the genera *Aeromonas*, *Aerobacter*, and *Acinetobacter*.

*Proteus* spp. bacteria isolated from the rhizosphere of rice in West Bengal, India, used hexachlorocyclohexane (HCH) pesticide [30] or phorate insecticide [31, 32] as a source of carbon and energy, and the addition of these chemicals to soil intensified the growth of bacteria. It is worth mentioning that *Proteus* spp. were the only members of the family *Enterobacteriaceae* isolated from these soil samples besides typical soil bacteria, actinomycetes, and fungi. Another example of autochthonous *Proteus* sp. strain, isolated from phorate-contaminated soil in India and able to degrade this insecticide, was described by Bano and Musarrat [11]. The strain exhibited also siderophore production, phosphate solubilizing capacities, and strong antifungal effect on phytopathogen *Fusarium oxysporum*, thus indicating the potential possibilities of its exploitation as PGPR as well.

The presence of *P. vulgaris* and *Proteus* sp. strains in microbial consortia capable of pesticide degradation was detected in highly contaminated soils in Colombia [116]. From soil samples exposed to different kinds of waste (including hazardous ones), a microbial consortium was isolated composed of ten strains, including *P. vulgaris* and *Proteus* sp. The consortium was able to degrade two widely used organophosphatic pesticides, chlorpyrifos and methyl parathion, both in culture medium and in soil. A similar bacterial consortium was found in highly contaminated soil samples from Moravia, Medellin, an area that was used as a garbage dump from 1974 to 1982 [115]. The consortium was able to degrade methyl parathion and p-nitrophenol as the only source of carbon and to decrease their toxicity in the medium and in soil at different depths.

Azo dyes frequently used for textile dyeing and paper printing and their metabolites present in effluents may be toxic, mutagenic, or carcinogenic. Their physicochemical neutralization is costly, while biological degradation is cost-effective and friendly to the environment, but difficult. For that reason, identification of the effective *Proteus* spp. degraders is a promising perspective for the environmental protection. Patil et al*.* [112] have isolated a *Proteus* sp. strain (related to *P. vulgaris*) from a waste site of the textile industry in India, which was capable of degrading seven textile azo dyes, but the most effective decolorization and detoxification of Navy Blue Rx were observed due to the induction of laccase and other enzymes. Chen et al*.* [25] isolated from sludge, obtained from a dyeing wastewater treatment plant in Taiwan, a *P. mirabilis* strain exhibiting a great capacity of efficient enzymatic reduction and decolorization associated with the biosorption of deep red RED RBN as well as deep black BK-5 azo dyes. Furthermore, Olukanni et al*.* [106] isolated from municipal dump site soil near Lagos, Nigeria, a *P. mirabilis* strain able to degrade a Reactive Blue 13 azo dye to phyto-non-toxic products. However, its laccase (the azo-dye-degrading oxidoreductase) activity was 70-fold lower than that of another very effective strain, *P. hauseri*, isolated from the Chiao-His hot spring in Taiwan [95, 157]. It effectively decolorized several mono- and di-azo dyes [23, 24] and was copper-tolerant as its laccase was a copper-induced enzyme with thermophilic and acidophilic properties [159]. However, the presence of copper in the environment reduced the swarming activity of the bacterium [96]. The isolate is the first reported non-pathogenic strain in the species *P. hauseri*, coming from natural water habitat.

There are more reports on the high tolerance of *Proteus* spp. bacteria to copper and other heavy metals. Ge et al*.* [45], from the East Sea (China) contaminated by chromate, isolated a *Proteus* sp. strain (related to *P. penneri* and *P. hauseri*) which was highly tolerant to Cr (VI) and could significantly reduce the amounts of this toxic metal in seawater. Hassen et al*.* [55] in Tunisia isolated from wastewater many Gram-negative bacteria with dominating *Pseudomonas* spp. and *P. mirabilis* strains, highly tolerant especially to copper and also to chromium, cobalt, cadmium, zinc, and mercury. Also, a highly resistant to several heavy metals (including mercury, copper, zinc, cadmium, cobalt, silver, and others) *P. mirabilis* strain was identified in wastewater samples from Casablanca City, Moroccco, also exhibiting resistance to naphthalene, anthracene, and antibiotics [40]. Another strongly copper resistant strain identified as *P. vulgaris* and isolated from soil samples collected near the Panki power plant in Kampur, India, was reported by Rani et al*.* [120]. The strain used to inoculate pigeon pea (*Cajanus cajan*) seeds protected the pea against the inhibitory effect of copper, reducing its amounts both in soil and in the plant. Moreover, the bacterium was able to produce siderophores providing pigeon pea with iron and preventing chlorosis.

There are more examples of soil *Proteus* spp. strains beneficial to plants (Table 3). Islam et al*.* [60] reported on a *P. mirabilis* strain highly resistant to zinc, found in agricultural field soil, irrigated with industrial effluents in Faisalabad City, Pakistan. The strain was able to block zinc absorption by maize (*Zea mays*) roots, reduce oxidative stress in the plant, and increase its tolerance to zinc, promoting maize growth in the presence of the heavy metal. Simultaneously, the bacterium enhanced phytoremediation processes conducted by this copper-resistant plant. Rau et al. [121] characterized a *P. mirabilis* strain isolated from rhizosphere of wild grass *Saccharum ravennae* colonizing fly ash dumps in Delhi, India. The strain displayed strong resistance to arsenic and was medium resistant to copper, chromium, cobalt, cadmium, zinc, mercury, nickel, and lead. Additionally, the strain produced siderophores with great capacity and was an active phosphate solubilizer, thus enhancing the growth of grass. Similarly, a *Proteus* sp. strain from a rice field in Pakistan inoculated on wheat seeds effectively solubilized rock phosphate composted with poultry litter, stimulating the plant growth [14]. Yet, rhizobacterial *P. vulgaris* strains, identified in acidic soil from a tea (*Camellia sinensis*) plantation in India, expressed siderophore production and antifungal properties both in acidic and in neutral pH conditions [12]. The strains successfully protected three tested species of leguminous plants from the pathogenic fungus *Fusarium moniliformae*; when the seeds were bacterized during the sowing, they were able to colonize roots and promote the growth of host plants. Lu et al*.* [76] found in cow dung in China *P. penneri* strains which produced volatile organic compounds killing within 1 h 98–100 % of tested nematodes: soil *Caenorhabditis elegans* and plant-pathogenic *Meloidogyne incognita*, thus expressing the possibility of being used as a protecting plant biocontrol agent. Yu and Lee [156] stated that indole production by the *P. vulgaris* strain (Table 1) isolated from the rhizosphere of halophyte glasswort (*Salicornia herbacea* L.) effectively promoted the growth of Chinese cabbage seedling (*Brassica campestris* ssp. *pekinensis*), while Bhattacharyya et al*.* [13] reported that the same strain stimulated the growth of *Arabidopsis thaliana* (a small flowering plant from the family *Brassicacae*) by indole production influencing the auxin, cytokinin, and brassinosteroid signaling pathways.

These promising results open wider possibilities of using specialized *Proteus* spp. strains as bioremediators and PGPR.

## Conclusion

Bacteria from the genus *Proteus* are well known as human opportunistic pathogens and intestinal microorganisms indicating fecal pollution of water or soil. Their presence in drinking water poses a threat of infection. On the other hand, the bacilli are believed to play an important role in removing organic pollutants of animal origin, especially fecal ones, by decomposing the dead organic matter in water or soil environments. Their proteolytic and ureolytic properties may place the bacteria among the most efficient saprobes taking part in the enrichment of manured soils in ammonia salts and therefore participating in the nitrogen cycle. However, the significance of these microorganisms in the mineralization processes of nitrogen-containing organic compounds is not obvious. This aspect of their metabolism requires more research. It would be reasonable to establish their exact contribution as destruents to the circulation of matter in nature.

*Proteus* spp. strains are sometimes found to co-operate with higher organisms, playing a role of animal and plant symbionts. Their antagonistic or commensal relationships with numerous animals have also been documented. However, environments other than the human body are less recognized as habitats of *Proteus* spp. rods. In the future, more attention should be paid to the interdependences between *Proteus* spp. and animals or plants coexisting with the bacteria in the environment. Relatively little information is available also about the mutual relations between *Proteus* spp. bacilli and other bacteria in natural habitats. Establishing what kind of relationships these are and which organisms benefit from this system is the basis for determining the reasons why the bacilli occur in a given ecosystem and the consequences of their activity.

Nowadays, molecular techniques provide proper tools to investigate the microorganisms in their natural habitats. The differences in the biochemical features do not exclude the strain from a genus as long as the similarities on the genome level are big enough. It is important to employ molecular techniques in the identification of *Proteus* spp. strains, especially those belonging to the new genomospecies, the biochemical differentiation of which is impossible. Metagenomic analysis of soil and water environments as well as of different niches in human, animal, and plant organisms using proper primer sequences may reveal the presence of *Proteus* spp. strains in these habitats even if they exhibit atypical activities and play unusual roles.

*Proteus* spp. bacteria often display exceptional metabolic features, allowing them to adapt to various conditions and reach distant niches in natural environments where they may be recognized as autochthons. Although the genus is usually not mentioned among PGPR or bioremediators, *Proteus* spp. environmental strains possess the abilities of immobilization of heavy metals, utilization of toxic pollutants, and plant growth stimulation, as well as nematicidal and fungicidal properties. Numerous examples suggest that the application of these microorganisms may be an effective way of protecting plants and groundwater. The biological treatment of polluted natural environments is more efficient and cost-effective than physicochemical methods and can be used in large areas due to simple application and an ability to remove pollution completely. Bioremediation could involve not only the stimulation of the natural autochthonic *Proteus* spp. strains but also introducing these microorganisms to the polluted environment.

However, it is important to remember that the bacteria belonging to the genus *Proteus* are opportunistic human and animal pathogens. It would be useful and interesting to compare the features (especially virulence factors, serotypes, metabolic apparatus, and the antibiotic resistance) expressed by *Proteus* spp. clinical isolates and by the strains existing in animal, soil, and water ecosystems. Do environmental *Proteus* spp. strains differ significantly from clinical ones? Do they possess genes coding the factors of pathogenicity identified in the strains isolated from patients? If so, do they express these factors and how do they use them in non-pathogenic conditions? What is the risk of transformation of a strain which could be used in bioremediation and environmental protection into a virulent one posing a threat to the animals or humans inhabiting the area? Do the environmental strains carry antibiotic resistance genes, which might lead to the transfer of these genes into other microorganisms in the environment? Answers to these questions could explain which factors contribute to and in what way they are important in different lifestyles presented by *Proteus* spp. bacteria in various environments.
